# Field and Laboratory Studies on the Ecology, Reproduction, and Adult Diapause of the Asian Comma Butterfly, *Polygonia c-aureum* L. (Lepidoptera: Nymphalidae)

**DOI:** 10.3390/insects9040169

**Published:** 2018-11-22

**Authors:** Satoshi Hiroyoshi, Gadi V. P. Reddy

**Affiliations:** 1 Laboratory of Applied Entomology, Tokyo University of Agriculture and Technology, Fuchu, Tokyo 183-8509, Japan; 2 202 Corpo Mankyu, 1-23-8 Nodamachi, Kawagoe, Saitama 350-1115, Japan; 3 Department of Research Centers, Western Triangle Agricultural Research Center, Montana State University-Bozeman, 9546 Old Shelby Rd., P.O. Box 656, Conrad, MT 59425, USA; reddy@montana.edu

**Keywords:** accessory gland, duplex, ovarian development, simplex, sperm, spermatogenesis, testis

## Abstract

Adult diapause and reproduction of a nymphalid butterfly, *Polygonia c-aureum* L., were investigated in field and laboratory examinations. Laboratory studies showed that old virgin male butterflies of non-diapausing generations had heavy accessory glands and simplex, which were suppressed in diapausing generations. The number of eupyrene sperm bundles in the duplex increased with adult age, whereas testis size decreased with age. Field examinations indicated that reproductive development of both sexes of diapausing generations in autumn was suppressed, and developed in spring. We attempted to estimate the physiological age of wild-caught males, as adult male age can be estimated from the testis size. We also attempted to determine whether or not wild male butterflies had mated from the development of the accessory glands and simplex, as well as the number of eupyrene sperm bundles in the duplex, by comparing unmated males with mated males. Field examinations suggest that almost all females in a population of non-diapausing generations mated and showed a tendency toward polyandry, while in the diapausing generation, in spring, monoandry rather than polyandry predominated. This suggests a different mating strategy between non-diapausing and diapausing generations.

## 1. Introduction

In general, field data on pest insects are abundant, and laboratory experiments reflect field data. On the other hand, having both field and laboratory data on non-pest species is less common, and the life histories of native insects are often poorly known. In the field, it is difficult to know the mating status of wild adult males apart from direct observation of mating, which is limited to species that have a long copula duration or where this behavior is more likely to be observed. Very often, laboratory studies may not reflect events (e.g., such as mating) as and when they would occur in the field. Understanding the reproductive development of adult males is important when considering male reproductive strategies.

Diapause is thought to be the mechanism allowing insect survival in the most severe climates [1,2]. In contrast to males, reproductive diapause in females including lepidopterans has been well studied [3,4]. Female adult diapause is characterized by a suppression of development of female accessory gland and of oogenesis, cessation of pheromone synthesis, and full development of the fat body [3]. For instance, in the ash leaf cone roller, *Caloptilia fraxinella* (Ely)*,* females exhibit prolonged reproductive diapause immediately after emergence in mid-summer, which lasts until the next spring, when mating occurs [5]. Although this finding might be applied to other adult diapausing lepidopteran females, a few species have been examined on their physiology. On the other hand, the relationship between spermatogenesis and adult diapause differs from species to species [6]. As to whether spermatogenesis is promoted or suppressed by environmental or genetic conditions, differences may exist within species or even life stages. For example, spermatogenesis or testis development is suppressed during the larval or pupal diapause in the codling moth, *Laspeyresia pomonella* (L.) (Lepidoptera) [7]; the sweet potato hornworm, *Agrius convolvuli* (L.) [8,9]; and the cabbage armyworm, *Mamestra brassicae* L. (Lepidoptera) [10], whereas spermatogenesis is active even during adult diapause of the sharpshooter *Draeculacephala crassicornis* Van Duzee (Hemiptera) [11]. In the anise swallowtail, *Papilio zelicaon* Lucas, sperm are produced continuously at a uniform rate in the adult stage [12]. However, adult spermatogenesis has been less investigated in the adult diapausing lepidopteran species. In the monarch butterfly, *Danaus plexippus* (L.), adults have a lot of mature sperm in the testis in the overwintered generation [13], but it is not known when spermatogenesis of the diapausing generation occurs. Thus, it is necessary to investigate the relationship between spermatogenesis and adult diapause to clarify when and why diapausing adult males produce and store the sperm in the duplex (sperm storage organ) for ejaculation.

Most male insects have only eupyrene-type sperm, which has a nucleus and thus can fertilize eggs. However, sperm polyphenism is known in several orders and species of insects [14]. In Lepidoptera, sperm diphenism, i.e., eupyrene (nucleate) and apyrene sperm (anucleate), is known [15,16,17], except for groups of primitive moths [18,19]. The function of apyrene sperm is unclear. For example, there is no evidence that apyrene sperm are involved in forming barriers between preexisting sperm and incoming eupyrene sperm from the subsequent mating within the spermatheca in the arctiid moth *Utetheisa ornatrix* L. [20]. However, the rapid decline in apyrene sperm in the spermatheca would support the hypothesis that they facilitate the movement of eupyrene sperm [21,22,23,24] or assist in the freeing of eupyrene sperm from the spermatophore [25]. It has been reported that the presence of apyrene sperm delays remating by female in the green-veined white butterfly, *Pieris napi* (L.) [26]. Spermatogenesis and sperm storage have not been examined in these insects, and thus we suppose that the results can be altered depending on the male age, mating history of both sexes, and environmental conditions.

Furthermore, in polyandrous lepidopteran species, the presence of many apyrene sperm in the spermatheca reduces the receptivity of the female to remating [27]. In the monandrous swallowtail butterfly *Byasa alcinous* (Klug), virgin males always transfer ejaculate that has a high proportion of apyrene sperm [28], as also occurs in some polyandrous species such as *Eureuma mandarina* (de I’Orza) [29] and *Papilio xuthus* L. [30]. It has been proposed that male investment in spermatophore quality is a function of the degree of polyandry, with the spermatophores of polyandrous species, such as *Heliconius* species, being lower in mass and protein content during second matings [31]. Males of the tobacco budworm, *Heliothis virescens* F., transfer large spermatophores into virgin females, but do not alter the ratio of eupyrene-to-apyrene sperm when mating with non-virgin females [32]. Moreover, males of this moth pass fewer sperm in the second ejaculate and older males transfer more sperm than do young males [32]. However, how adult apyrene or eupyrene spermatogenesis relates to mating strategy is still unclear.

The development of adult male reproductive organs in Lepidoptera is less understood. One exception, the monarch butterfly has been thoroughly studied. In this butterfly, the development of male accessory glands and simplex is suppressed during adult diapause, although during the non-diapausing generations, these organs (used for spermatophore transfer) do develop [33,34]. Although this butterfly does not exhibit seasonal polyphenism, the autumn generations famously undergo long distance migrations to Mexico, California, and Florida [13,35,36,37,38]. After adult diapause, they migrate from these overwintering areas toward the northern United States and Canada [39]. The role of male accessory glands secretion is thought to ensure reproductive success by either facilitating transfer of sperm or providing sperm protection, although in a number of species, the accessory glands may serve many roles [40]. In *D. plexippus*, a sperm activator substance is contained in the simplex [41]. The mechanism controlling development of accessory glands and simplex is probably dependent on the activity of corpora allata, which is a juvenile hormone [42].

Assessing whether adult females mate or not is easy, except for in the cases of incomplete or failed matings [43]. In Lepidoptera, the main body of spermatophores consists of the contents of the simplex. In *D. plexippus*, a spermatophore represents approximately 10% of body mass [44]. In addition, no more than one spermatophore is formed during a single mating, because a certain minimum time is required for spermatophore construction [45]. Thus, an extremely short copula duration by accident would provoke mating failures. In Lepidoptera, spermatophore(s) often break down or collapse, the time frame for which seems to vary by species from several hours to several days [46,47]. In females, the presence of a spermatophore is an indication of recent mating. For this reason, the number of successful matings can be ascertained by dissecting the bursa and counting spermatophores present [48]. Currently, it is considered that females of many Lepidoptera are polyandrous [49,50], although there are monandrous species [48] and the degree of polyandry varies greatly among species [51,52,53,54,55]. Consequently, females may re-mate with males depending on conditions such as mating interval, age, size of spermatophore, or the presence or quantity of sperm [56,57]. Understanding the factors regulating polyandry is important in studying the evolution and adaptive significance of mating systems [58].

Accessory gland substances that are transferred into females at mating have various functions in reproduction for both sexes. It is presumed that these secretions in insects are involved with insemination, such as sperm transfer, sperm activation, mating plugs and spermatophragma, insemination reaction, modification of copulative behavior, contributions to survival and fecundity, inhibition of receptivity, sperm storage in female’s reproductive tract, antibacterial activity, the influence on egg maturation and oviposition, or cessation of pheromone release or production [59,60,61,62,63,64,65,66,67,68,69,70,71]. Although multiple matings often increase these functions, it may risk the contraction of physical injures by males, predation, a sexually transmitted disease or some other sterility-inducing factor [72,73]. It is important to investigate the development of accessory glands and simplex to understand adult diapause of males, because the development of these organs is related to mating ability, which most probably is suppressed during the diapause.

The Asian comma butterfly, *Polygonia c-aureum* L., the object of our study, is polyvoltine in Japan, except for cold northern regions, and this butterfly shows distinct seasonal diphenism, i.e., summer and autumn forms, with respect to the shape and coloration of wings and in the butterfly’s reproductive activity. Summer-form (non-diapausing) butterflies emerge in summer and begin to reproduce shortly after emergence. The wings of the summer-form are lighter in color with larger dark spots and shallower notches in wing margin than the autumn-(diapausing) form. Autumn-form butterflies appear in autumn, enter diapause after adult eclosion, and do not reproduce until the following spring. Seasonal form and adult diapause are determined mainly by the photoperiod and temperature conditions experienced by larvae [74,75]. Diapause in *P. c-aureum* female is maintained for a month under a short photoperiod at 20 °C, but is terminated by a long photoperiod [76]. Butterflies feed on the nectar of the various flowers, whereas larvae eat leaves of *Humulus japonicas* Siebold & Zucc (Cannabaceae) mainly.

Sperm storage in lepidopteran males is often called sperm movement. It is known that sperm movement from the testis to the duplex via the paired vasa deferentia occurs daily in Lepidoptera [77], and thus sperm accumulate in the duplex and increase in number with age [78]. This rhythm disappears when the lepidopteran insects are kept under continuous light, but the pattern returns after transfer to alternating light and dark cycles [79,80], although constant light has no adverse effects on the sperm movement of the codling moth, *C. pomonella* [81]. Upper vas deferens has cyclical secretory activity by the epithelium of the anterior segment in correlation with the circadian rhythm of sperm release from the testes [82]. It has been demonstrated in vitro that daily sperm movement is governed by a circadian clock [83]. Such a circadian rhythm is entrained by the photoperiod and has been demonstrated in several moth species [84,85,86]. As a result, the weight of the duplex increases with adult age, because sperm accumulate daily, resulting in an increase in weight of the vas deferens and duplex. In fact, the weight of the vas deferens increases between the emergence day and examination day in *D. plexippus* [87], and the number of eupyrene sperm bundles in the duplex increases with adult age in *P. c-aureum* [78]. These results imply that unmated old lepidopterans have many sperm in the duplex, whereas unmated young ones have a few sperm.

The testis of *P. c-aureum* starts to shrink at the end of the pupal stage [88]. Testis shrinkage has been shown in various lepidopteran species, e.g., *H. virescens* [89], *Helicoverpa assulta* (Guenée) [90], the Japanese oak silkworm (*Antheraea yamamai* Guérin-Méneville) [91], *Trichoplusia ni* (Hübner) [92], *Spodoptera litura* (Fabricius) [93], *Ostrinia nubilalis* (Hübner) [94], *Manduca sexta* (L.) [95], *L. dispar* [96], *Boarmia selenaria* Schiffermiller [97], *P. xuthus* [98], and *D. plexippus* [87]. Since the testis shrinkage of *P. c-aureum* continues to the adult stage, we hypothesize that testes size can be used to estimate the physiological age of field-caught male butterflies [88]. If physiological age, ideally exact age, can be estimated, it may be possible to determine whether or not wild adult males had mated by comparing the reproductive development of unmated and mated adult males under laboratory conditions. Alternatively, we need to compare this method with generally adopted methods on wing wear to estimate the physiological age of wild adult males.

In this study, we first presented field data and laboratory data on both sexes of reproduction and adult diapause of *P. c-aureum*. Additionally, whether the wild female butterflies mated or not was examined in various season. We then tested whether estimation of physiological age and mating status in wild-caught male butterflies was possible. Lastly, we described the phenology of this species and their larval host plant.

## 2. Materials and Methods

### 2.1. Insects

*Polygonia c-aureum*, the species used in this study, is distributed across Japan, the Korean peninsula, Taiwan, China and the Indochina peninsula. In Japan, their apparent distribution in the northern island of Hokkaido is limited to the southwest regions, probably due to a lack of host plants or extreme low temperatures in winter. They are likewise not found on Okinawa and Amami islands, in the extreme south of Japan, although they are often observed on Okinawa and other neighboring islands in the autumn only, where their host plant is absent, probably having migrated from other islands, such as Tanegashima Island (to the northeast) or Taiwan (southwest from Okinawa).

### 2.2. Rearing Methods

The *P. c-aureum* colony used in this study derived from larvae collected in Tokyo and Saitama Prefectures in 1989 was successively reared in the laboratory at 21 ± 1 °C and a long day (15:9 L:D) photoperiod. Adults of reproductive generations consist of groups of approximately 100 butterflies containing both males and females to get offspring used in the experiments. Only individuals from eggs laid by summer-form adults were used for experiments, because the offspring of autumn-form females are likely to become the summer-form under any photoperiodic conditions due to a maternal effect [99].

Larvae were reared in groups of 30 to 40 on pieces of filter paper in glass Petri dishes (12 cm dia × 3 cm depth or 15 cm dia × 4 cm depth) on fresh leaves of Japanese hop, *Humulus japonicas* Siebold & Zucc. After pupation, pupae were transferred into polythene bags with soft tissue paper to absorb the meconium and to facilitate normal adult eclosion. Immature stages were reared under a short day length (8:16 L:D) photoperiod at 21 ± 1 °C or a long photoperiod as mentioned above. After adult emergence, females and males were kept in separate cages (17 × 16.5 × 46 cm) covered with Saran® net in groups of 30 to 40 individuals to prevent mating. Adults were fed a 10% sugar solution on cotton. In the experiments to assess reproductive developments under laboratory conditions, all butterflies of both sexes were virgin.

### 2.3. Field Examination

Field examinations were performed mainly at Akigase Park (35.51 °N, 139.36 °E), Urawa, Saitama, close to Tokyo. Examinations were made monthly (except in May and winter) in 1991 and 1992, because we could not find any butterflies during local winter (December–February). In 1991, a study field was flooded by a typhoon, and we therefore repeated one field survey in 1992. We tried to catch as many butterflies as possible except for October, as there were too many to dissect all present in October. Thus, the population size in October was much higher than that when most of the data of this study were collected. Butterflies collected in the field were brought to the laboratory alive, where they were kept into the refrigerator at 5 °C until use.

### 2.4. Experimental Protocols

#### 2.4.1. Measurement of Forewing Length

Forewing length was measured in all individuals when possible, although lengths of broken forewings could not be measured. Forewing length might be correlated to the pupal weight or adult size, because pupal weight under SD is lighter than those under LD when the larvae were reared under various photoperiodic conditions [100].

#### 2.4.2. Diapause Status

To assess diapause status of butterflies, butterflies reared under laboratory conditions and butterflies immediately after collection in the field were dissected to examine the development of reproductive organs; in females, ovarian development and the presence or absence of spermatophores were assessed as mentioned below, while in males, reproductive organ development such as testis, accessory glands and simplex were examined. Female butterflies with greenish or green oocytes were considered to be non-diapausing, whereas butterflies with transparent or white oocytes were considered to be in diapause. In males, diapause is defined to have underdeveloped accessory gland and simplex [42], which produce spermatophoral substances and not transferred to females due to suppression of mating. Mating occurred mainly during the earlier end of the latter half of the photophase under laboratory conditions at 21 °C. In the field, copulative behavior can often be observed in the afternoon, but we could not find any mating pairs, because they mate beneath the host plant.

#### 2.4.3. Testis Size

The width (W) and length (L) of the testis were measured with the aid of a calibrated ocular micrometer under a phase-microscope. Testis volume was calculated using the following formula: Volume = 4/3π × W^2^ × L, assuming a spherical or elliptical shape. Measurement of testis size was performed in all insects reared in the laboratory and caught in the field, since it is used to estimate age and to compare with other reproductive characteristics.

#### 2.4.4. Development of Accessory Glands and Simplex

To determine the development of the accessory glands and simplex, those organs were kept in the desiccator for three days or longer. Their dry weights were measured in groups of five virgin individuals to the nearest 0.1 mg by an electronic balance every 10 days after adult emergence. Dry weight of those organs in wild-caught butterflies was also measured individually. Substances from the simplex made up most of the spermatophore in Lepidoptera [40]. Semen was present inside spermatophores, including sperm and white substances derived probably from the accessory glands, testis, vasa deferentia, and duplex.

#### 2.4.5. Spermatophore

Dead females of reproductive generations reared successively were dissected to find out how many times they mated by counting the number of spermatophores in the bursa copulatrix. We also counted the number of spermatophores in butterflies collected in the field. Volume of spermatophore was also measured by the formula used for the testis size as mentioned above.

#### 2.4.6. Mating Experiments

For mating experiments, emerged butterflies of both sexes were separately reared in adult cages as mentioned above, and 10 virgin females and 10 virgin males were introduced into this cage during the latter half of photophase. Mated females were then dissected to measure the size or dry weight of spermatophores, and assess the presence of sperm in the seminal receptacle. Females that did not copulate for three consecutive days were replaced by other virgin females. Males were paired with virgin females every day for the first 30 days of their life, and the number of matings counted. Dry weight of spermatophores kept in the desiccator for three days or longer was measured in each mating per male butterfly as mentioned above. Volume of spermatophores was also measured by the formula used for testis size. The effects on the spermatophore size of virgin male age at first mating, the number of matings, ranging from 0 to 15, and the interval (days) between matings, ranging from 1 to 17, were examined for each male butterfly.

#### 2.4.7. Spermatogenesis

To investigate the process of spermatogenesis quantitatively, larvae and pupae were dissected every day after 4th instar larval molt, and virgin butterflies reared under LD or SD photoperiodic conditions in their entire life span were dissected every 10 days, from emergence to day 30 of adult life. Mated or virgin butterflies collected from the field were also dissected for comparison. Testes, which are fused at the prepupal stage [101], were dissected out in a saline solution (8.6 g NaCl, 0.33 g CaCl_2_ and 0.1 g KCl per liter distilled water) and ruptured with forceps to release the spermatocysts (cysts). The number of cysts was then counted under a phase-contrast microscope and categorizing them according to their size and type, as defined below. The cysts were divided into six classes based on the size, morphology, and development. Spherical cysts were divided in two size classes: small (36–85 μm in diameter) and large (>85 μm). Small or large cysts were further divided into three developmental phases, i.e., spherical, pyriform and elongate cysts. As cysts increased in size, some small spherical cysts developed to large eupyrene spherical cysts. Spherical cysts less than 36 μm in diameter were not recorded, because they were too numerous in the larval and pupal stages to count and could hardly be distinguished from other cells separated from broken cysts. The number of small spherical cysts was thus underestimated. It was easy to distinguish large pyriform and elongate cysts from small pyriform and elongate cysts, respectively, since the former were approximately twice as long and thick than the latter. In general, lepidopteran eupyrene and apyrene sperms have the following three features: (1) eupyrene sperm is far larger than apyrene sperm [92,102,103] except for in *S. litura* [104], (2) apyrene sperm bundles dissociate into single sperm, while eupyrene sperms remain as bundles when they move from the testis to the post-testicular organs [105], (3) eupyrene spermatogenesis precedes apyrene spermatogenesis [106,107,108]. Since our preliminary examination indicated that all the above features could be found in *P. c-aureum*, large spherical, pyriform or elongate cysts were considered as stages destined to become eupyrene sperm, and small spherical (except some cysts as mentioned above), pyriform or elongate cysts were expected to become apyrene sperm.

### 2.5. Phenology of Host Plant

We observed the development of the key host plant of *P. c-aureum* at the Tokyo University of Agriculture and Technology, Fuchu site and at other sites in Tokyo Prefecture, as well as in the Akigase Park of Saitama Prefecture, Japan.

### 2.6. Statistical Analysis

When sample sizes were too small or too unbalanced to use the normal distribution to compare two experimental populations, the non-parametric Mann–Whitney *U* test was used to analyze the data on forewing, spermiogenesis, or sperm storage in the duplex. Percentages were analyzed by goodness of fit test. Samples divided into three or more categories were analyzed by Tukey’s method after ANOVA.

## 3. Results

### 3.1. Forewing Length

The data on the forewing length was totalized and compared between summer- and autumn-form in both sexes, and between laboratory reared and field-caught male butterflies in both seasonal forms by Mann-Whitney *U* test. The forewing length (average ± S.D.) of the autumn-form wild adult males (26.35 ± 1.18, n = 43) was significantly shorter than that of the summer-form wild adult males (28.69 ± 1.40, n = 102) (*U1*-Value = 374.0, *U2*-Value = 4012.0, *W*-Value = 1320.0, *p* = 0.0000). The forewing length of autumn-form adult females butterflies caught from the field in 1992 (26.71 ± 1.49, n = 31) was significantly smaller than that of summer-form butterflies (28.95 ± 1.53, n = 69) (*U1*-Value = 99.5, *U2*-Value = 613.5, *W*-Value = 889.5, *p* = 0.0000). On the other hand, forewing length of laboratory reared autumn-form male butterflies (23.32 ± 1.21) was significantly shorter than that of field-caught ones (26.35 ± 1.18) (*U1*-Value = 126.0, *U2*-Value = 2841.0, *W*-Value = 3787, *p* = 0.0000). Similarly, the forewing length of laboratory reared summer-form male butterflies (26.29 ± 1.64) was significantly shorter than that of field-caught summer-form male butterflies (28.92 ± 1.34) (*U1*-Value = 742.0, *U2*-Value = 5378.0, *W*-Value = 2572.0, *p* = 0.0000). If the reproductive organs have a correlation with a body size, it should be considered about the differences of size and weight of reproductive organs.

### 3.2. Diapause

Adult diapause of female *P. c-aureum* butterflies is characterized by suppression of oogenesis and reproductive accessory glands [109,110]. In males, the development of accessory gland and simplex is suppressed under diapausing conditions (a short photoperiod and lower temperature) [42]. In addition, copulation is suppressed in diapausing butterflies of both sexes [111], and all female butterflies used in this experiment were virgin. Under short day length, ovarian development was completely suppressed during the first 30 days after emergence, but thereafter developed with age (Table 1). On the other hand, oogenesis developed with increased adult age if butterflies were kept under a long photoperiod after adult emergence (Table 1). These results indicate that photoperiod experienced during the adult stage also affected reproductive diapause in this butterfly.

Summer-form butterflies collected in the field progressed their ovarian development and almost all of them mated, as determined by the spermatophores in their bursa copulatrix (Table 2). In contrast, autumn-form butterflies caught in autumn showed suppressed oogenesis and mating receptivity (Table 2). Since we found that those autumn-form butterflies that were later caught in spring had mated, it is clear that oogenesis eventually progresses.

### 3.3. Spermatophore and Mating in Females

Wild summer-form females collected on 4th September were dissected to determine the relationship between the number of matings and spermatophore volume size. We found that the one-time mated females had larger spermatophore than twice mated females (Standard error = 7.9757, z-Value = −3.558, *p* = 0.00188), while twice mated females had smaller spermatophores than thrice mated females (Standard error = 9.2715, z-Value = 3.342, *p* = 0.00412) (Table 3), suggesting that mating frequency of females might affect the spermatophore size.

We presented here the results on mating frequency of females (data not shown). The number of spermatophores ranged from 0 to 4 in summer-form female butterflies reared in the laboratory for reproduction (1.422 ± 0.974, mean ± S.D.). The number of spermatophores in wild-caught summer-form butterflies (2.080 ± 1.222) that ranged from 0 to 4 was significantly greater than that in the laboratory-reared summer-form butterflies (n = 116, 1.422 ± 0.974) (*U1*-Value = 1090.0, *U2*-Value = 2042.0, *W*-Value = 2420.0, *p* = 0.0085). There were no significant differences in the number of spermatophores in the bursa copulatrix between autumn-form butterflies caught in spring (n = 10, 1.400 ± 0.699) and summer-form ones caught in summer (n = 25, 2.080 ± 1.222) (*U1*-Value = 88.0, *U2*-Value = 162.0, *W*-Value = 143.0, *p* = 0.1405). 

Next, the weight and/or volume of spermatophores were investigated in relation to the order of mating (Table 3). The spermatophore in the bursa copulatrix nearer the vagina is considered to have been transferred in the most recent mating, and the spermatophores were then numbered in the order of mating. The volume of the most recently deposited spermatophore varied in relation to number of matings, and spermatophores in autumn-form butterflies that were caught in spring (n = 6, 53.92 ± 24.64) were not different from those in summer-form butterflies caught in summer (n = 24, 33.56 ± 20.72) (*U1*-Value = 104.0, *U2*-value = 40.0, *W*-Value = 124.0, *p* = 0.0971). There was no significant difference in spermatophore volume of the last mating between autumn and summer forms (*U1*-Value = 71.0, *U2*-Value = 49.0, *W*-Value = 86.0, *p* = 0.5557). The volume of spermatophores in the wild summer-form was similar among those from different mating numbers (Table 3). The rate of re-mating of overwintered autumn-form females was 30% (n = 10), and the mating rate was 100%. On the other hand, the re-mating rate of non-diapausing summer-form females was 52% (n = 25) and the mating rate was 96%. The shape of spermatophores from the first, 2nd, 3rd and 4th matings was usually distorted. Thus, data from older spermatophores in this study may be affected if the spermatophores in the bursa copulatrix were collapsed or had disappeared or a mating failure had occurred.

### 3.4. Male Spermatophore Production Ability

When the effects of male mating frequency on weight or volume of spermatophore in *P. c-aureum* were examined for the first 30 days of adult life (Table 4), numbers of matings ranged from 0 to 15, and averaged 5.11 ± 4.8. The mean (± S.D.) dry weight and volume of spermatophores per mating was 1.93 ± 0.46 mg and 59.7 ± 48.7 mm^3^, respectively. A few individuals mated more than 10 times.

**Figure 1:**
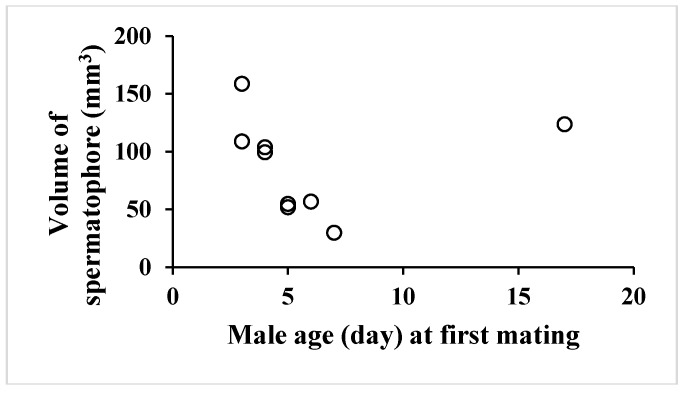
A correlation between volume of spermatophore and adult male age at first mating in summer-form male butterflies of *Polygonia c-aureum*.

**Figure 2:**
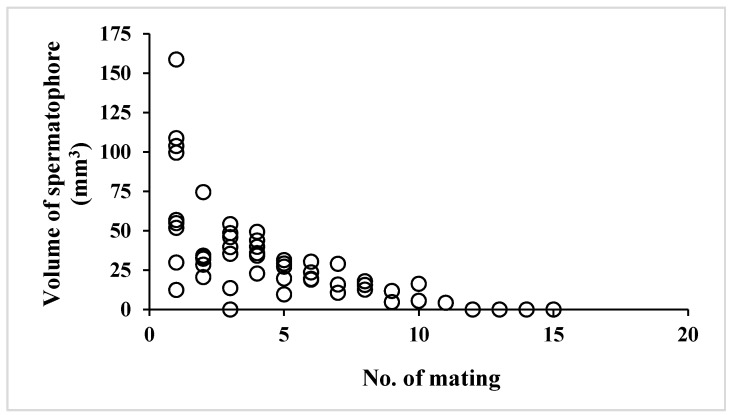
A correlation between volume of spermatophore and the number of mating in summer-form male butterflies of *Polygonia c-aureum*.

**Figure 3:**
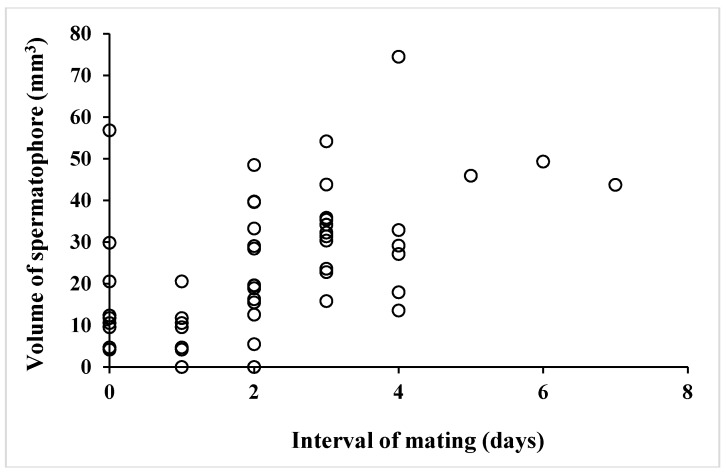
A correlation between volume of spermatophore and interval of mating (days) in summer-form male butterflies of *Polygonia c-aureum*.

Total volume of spermatophore differed in individuals greatly (Table 4). Interestingly, dry weight and volume of spermatophores decreased with mating frequency (data not shown), indicating a limited capability to produce more spermatophores. There was no correlation between adult male age at first mating and spermatophore volume (Figure 1) (y = 0.499147x + 84.568676, r^2^ = 0.0026941, *p* = 0.8945), while a negative correlation between male mating frequency and spermatophore volume (Figure 2) (y = −5.226726x + 59.209433, r^2^ = 0.394714, *p* < 0.0001). These results suggested that age of virgin adult males did not affect the size of spermatophores and that spermatophore size decreased with mating frequency. Finally, a positive correlation was found between spermatophore volume and interval (days) between matings (Figure 3) (y = 5.614266x + 11.3460127, r^2^ = 0.269841, *p* < 0.0001), which suggests that male butterflies can revive spermatophore production in the simplex after mating to some degree if given time to do so.

### 3.5. Development of Male Reproductive Organs

We investigated testis development under both field and laboratory conditions. In an earlier study, we suggested that adult male age can be estimated from testis size in lepidopteran insects [88]. Because the testis starts to decrease in size from the middle or late pupal stage, depending on the species, and continues to decrease in the adult stage, the age of wild adult males can be estimated from the testis size, irrespective of mating experiences, at least in species with long longevity. From the mating experiments as mentioned above, there was no correlation between mating frequency and testis size (y = 0.143362 + 18.209983, r^2^ = 0.100704, *p* = 0.2907), indicating that mating frequency did not affect the testis shrinkage (data not shown). Thus, it seems highly likely that mating appears not to affect testis development.

Field data showed that testis volume was small (5.490 ± 0.950 mm^3^) in autumn-form butterflies in late autumn (November) and also in March after overwintering (2.95 ± 1.493) and while testis volume was large (12.89 ± 4.079 and 13.13 ± 2.505) earlier in autumn (September and October), respectively (Table 5). On the other hand, summer-form butterflies showed relatively large testis during the summer, which later decreased in size after 4 September (Table 5).

**Figure 4:**
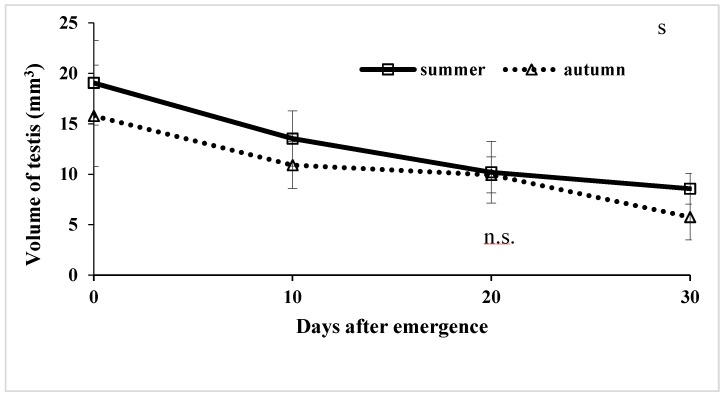
Comparison of testis volume (mean ± SD) with each adult age between summer- and autumn-form male butterflies of *Polygonia c-aureum* under laboratory conditions. Mann–Whitney *U* test was used for analysis. N.s. indicate no significant difference.

Testis size of virgin adult males was also examined under laboratory conditions to control for the effects of photoperiod. Under laboratory conditions, testis of both autumn-and summer-form butterflies decreased in size with adult age, regardless of photoperiod (Figure 4). These results indicate that adult males with small testis are old, whereas those with large testis are young. Summer-form males had significantly larger testis than autumn-from males at any age except for day 20 (n = 23, *U1*-Value = 89.0, *U2*-Value = 37.0, *W*-Value = 82.0, *p* = 0.1012), probably due to the differences of body size between autumn- and summer-forms: summer-form butterflies are larger than autumn-form butterflies as mentioned above.

We investigated the status of both the accessory glands and the simplex under both field and laboratory conditions. Field data indicated that there was a change in the weight of accessory glands in autumn-form butterflies across the season. Interestingly, one autumn-form male collected on the 4th of September had a heavy accessory gland, suggesting that he might not enter adult diapause.

Male autumn-form butterflies collected in autumn (late September to November) before overwintering tended to have lighter accessory glands (0.2, 0.13, or 0.20 mg, respectively), than those collected in spring (March and April) after overwintering (0.8, 0.48, or 0.39 mg, respectively) (Table 5). On the other hand, the summer-form wild-caught butterflies showed fluctuated changes in the weight of accessory glands over the season (Table 5). This is likely due to mating reducing the weight of accessory glands.

**Figure 5:**
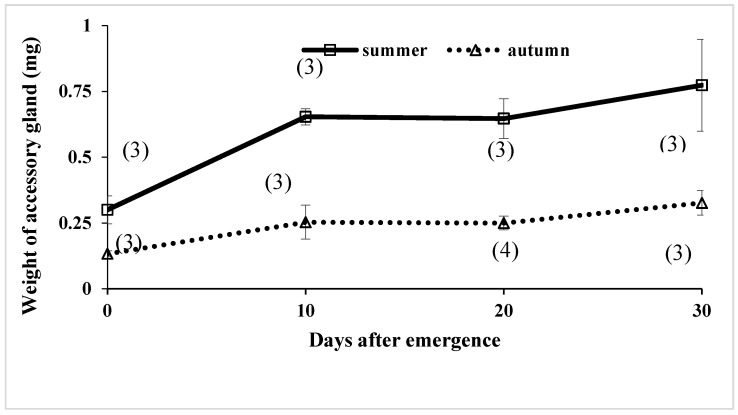
Comparison of weight of male accessory glands with adult age between summer- and autumn-form virgin male butterflies of *Polygonia c-aureum* under laboratory conditions. The number of parenthesis indicates the number of measurements repeated.

The weight of accessory glands was compared between the summer-form virgin butterflies kept under a long day-length at 21 °C in their entire life and the autumn-form virgin butterflies kept under a short day-length at 21 °C (Figure 5). Sample size was too small (usually just three replicates) to analyze the data, but in general, summer-form butterflies showed higher accessory gland weights than autumn-form butterflies. It is noted that this difference was noted immediately upon emergence, indicating that the development of accessory glands starts during the pupal stage.

Field data showed the changes in weight of the simplex (Table 5). In the autumn-form butterflies, the simplex was light (1.90 or 2.15 mg, respectively) in autumn before overwintering, whereas it was relatively heavy (4.00, 3.13, or 2.65 mg, respectively) in spring after overwintering (Table 5). The weight of simplex in the summer-form fluctuated greatly across the season (Table 5). A light simplex seemed be either from young age and/or mating.

**Figure 6:**
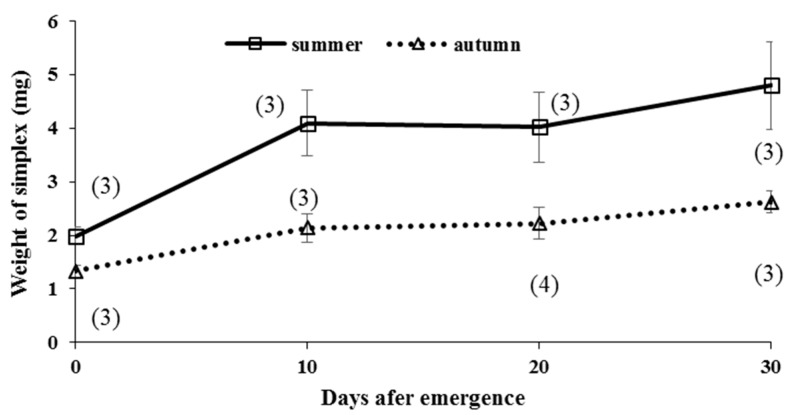
Comparison of weight of simplex with each adult age between summer- and autumn-form male butterflies of *Polygonia c-aureum* under laboratory conditions. The number of parenthesis indicates the number of measurements repeated.

The laboratory study showed distinct differences in the weight of the simplex between summer- and autumn-form virgin butterflies (Figure 6). Similar to the results for the accessory gland, short photoperiods suppressed the development of the simplex, while long pupal and adult photoperiods promoted it both after and before adult emergence, although the sample size was too small to analyze statistically. We observed that the weight of the simplex reduced greatly after several matings.

### 3.6. Spermatogenesis

**Figure 7:**
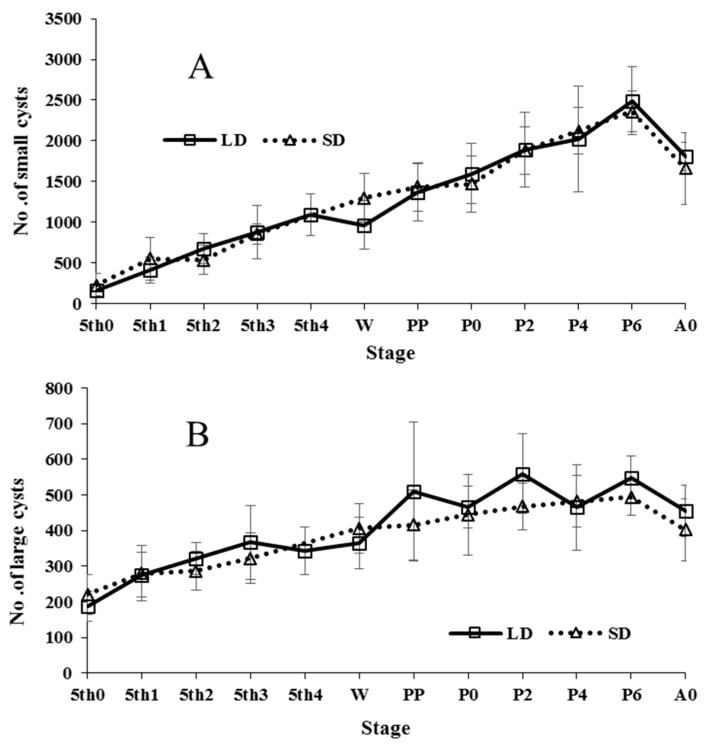
Comparison of larval and pupal spermatogenesis of small (**A**) and large (**B**) spermatocysts of *Polygonia c-aureum* between LD (long day-length) and SD (short day-length) under laboratory conditions. Sample size was approximately 10 at each point.

Spermatogenesis was compared between larvae and pupae reared under long or short photoperiod, in both small (Figure 7A) and large cysts (Figure 7B). We pooled the data on the changes of cysts in the spherical, pyriform, and elongate types for either small or large forms, since each type (spherical, pyriform, or elongate) of cyst showed similar tendencies [112]. Photoperiod had almost no effect on spermatogenesis in immature-stage insects of either individuals destined to become the non-diapausing summer-form or becoming diapausing autumn-form butterflies. The number of cysts increased progressively from 5th instar larvae to day 6 after pupation, and thereafter started to decrease through the remainder of the pupal stage.

**Figure 8:**
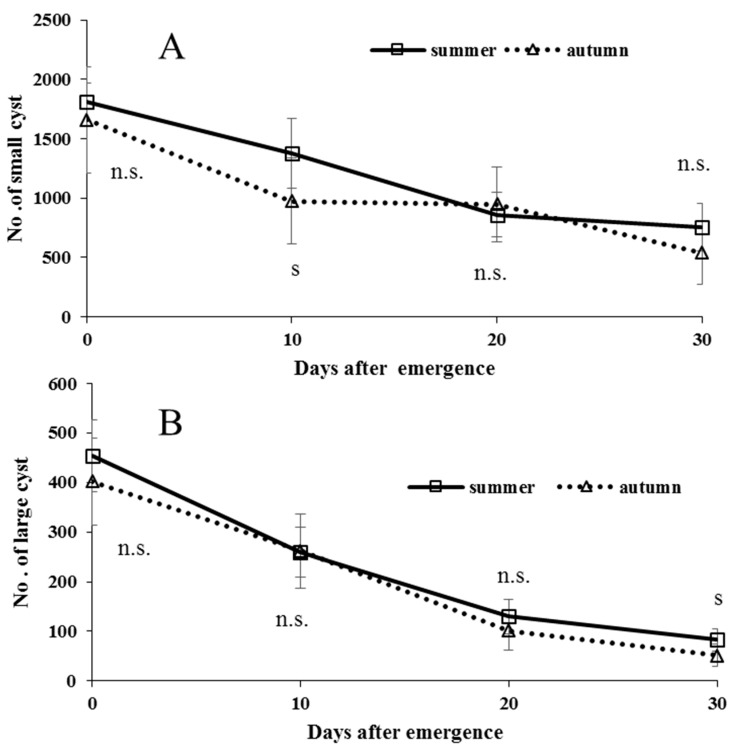
Comparison of adult spermatogenesis in small (**A**) and large (**B**) spermatocysts between summer- and autumn-form male butterflies of *Polygonia c-aureum* under laboratory conditions. Sample size was approximately 10 at each point. S and n.s. indicate the significant difference and no significant difference, respectively.

After emergence, summer-form butterflies were reared under long photoperiod, whereas autumn-form butterflies were reared under short photoperiod, both respectively, at 21 °C (Figure 8A,B). These adult males used were all virgins. The number of both small and large cysts, in both seasonal forms of the butterfly, decreased with adult age. Adult apyrene spermatogenesis was relatively active in young adults, whereas eupyrene spermatogenesis was inactive especially in old adults. It is noted that there are no evidences in any insects that mating enhances spermatogenesis to compensate the sperm loss used for ejaculation.

**Figure 9:**
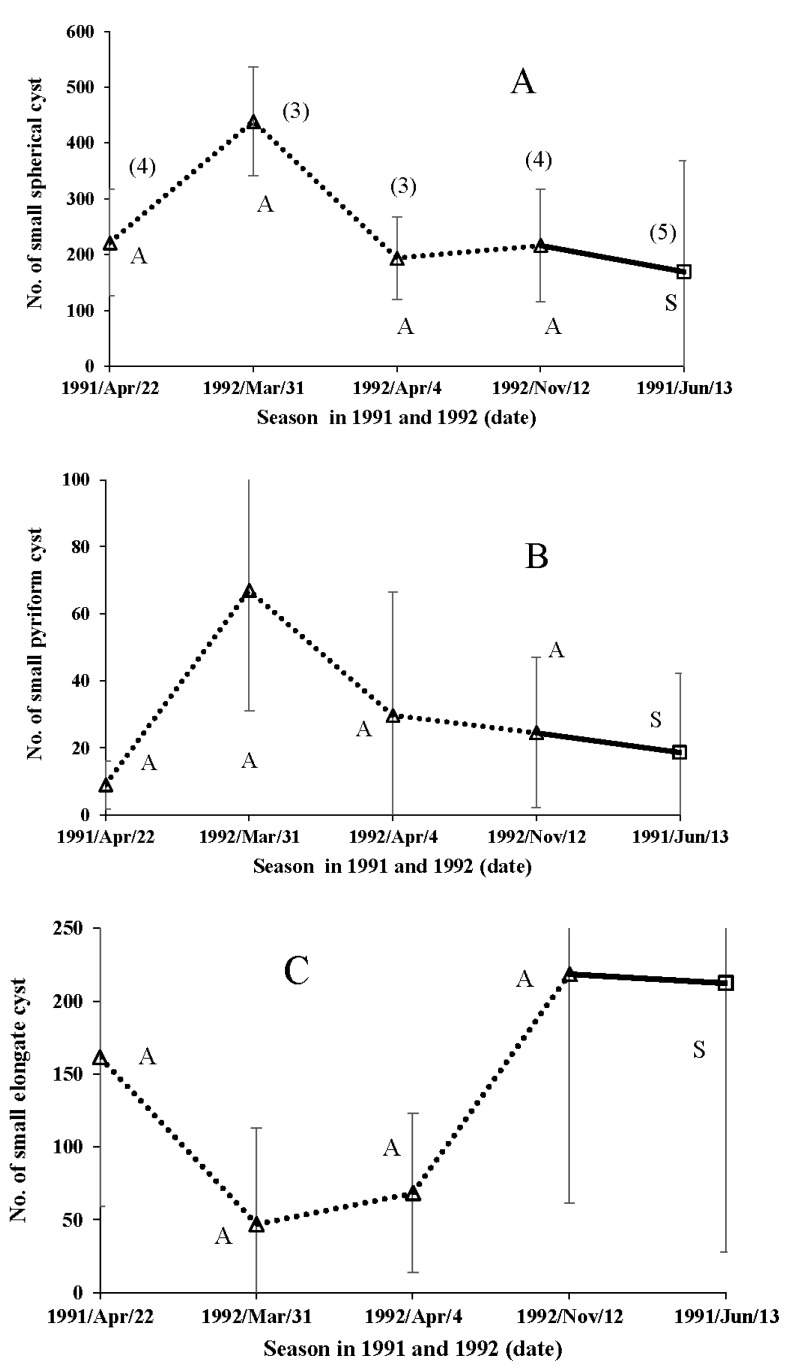
Field data on adult spermatogenesis in small spherical (A), pyriform (B) and elongate (C) spermatocysts over various season in the wild male butterflies of *Polygonia c-aureum*. Open square with solid lines and open triangle indicate the results of summer-form (S) and autumn-form (A), respectively.

**Figure 10:**
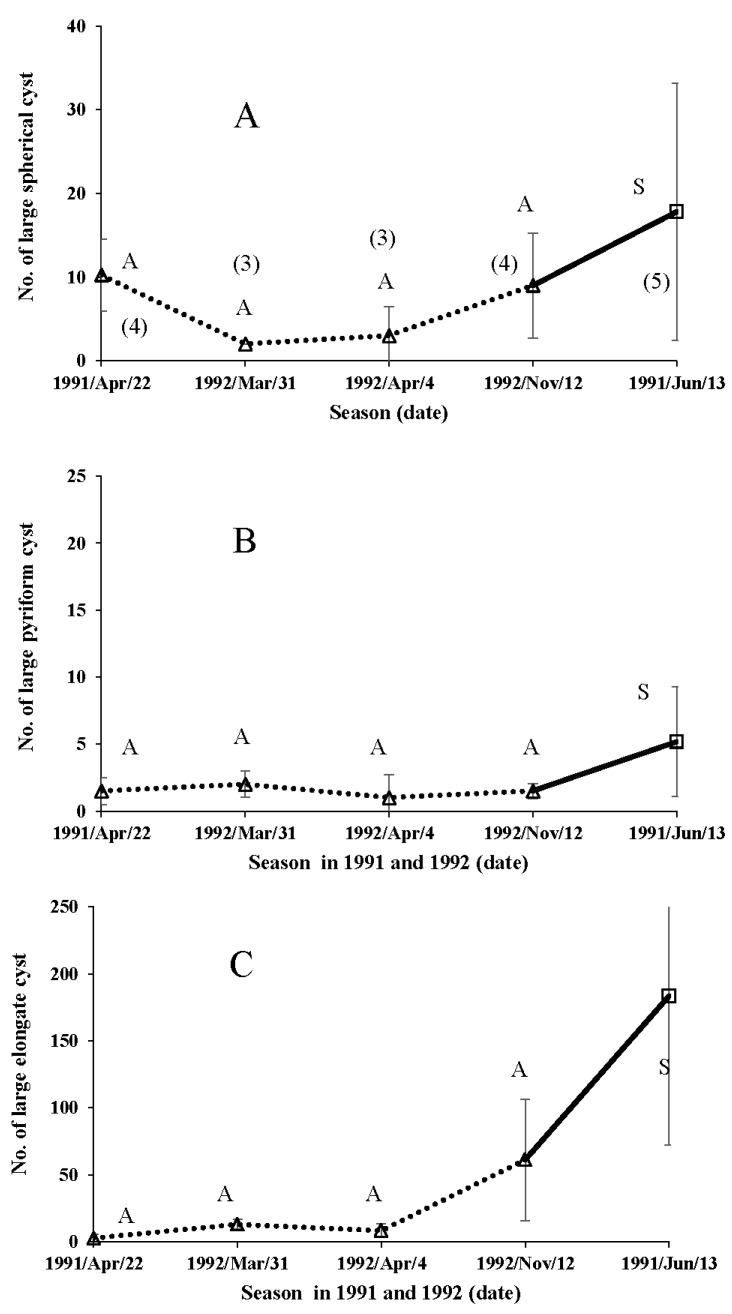
Field data on adult spermatogenesis in large spherical (A), pyriform (B) and elongate (C) spermatocysts over various season in the wild male butterflies of *Polygonia c-aureum*. Open square with solid lines and open triangle indicate the results of summer-form (S) and autumn-form (A), respectively. The number of parenthesis indicate the sample size, which was the same with other results (**B**,**C**).

In the field examinations, the number of small spherical cysts varied between approximately 200 and 450 (Figure 9A), indicating the relatively intense apyrene spermiogenesis ability in both seasonal forms of the butterfly in any season. It was remarkable that the number of small pyriform cysts was lowest in overwintered male butterflies (Figure 9B). The number of small elongate cysts in male butterflies collected in autumn and summer was over 200 (Figure 9C). There were only a few large spherical cysts (eupyrene sperm) on any of the sample dates, in butterflies of either seasonal form (Figure 10A). Similarly, the number of large pyriform cysts was extremely small throughout the year, again for both seasonal forms of the butterfly (Figure 10B). Moreover, the number of large elongate cysts was very small in the overwintered autumn-form male butterflies (Figure 10C), although our earlier study suggests an increase in the production of eupyrene sperm after overwintering [113].

### 3.7. Sperm Movement

Sperm movement implies the release or transfer of sperm from the testis to the vasa deferentia and then from vasa deferentia to the paired duplex. Field examinations showed that the number of eupyrene sperm bundles in the duplex was relatively high in autumn-form adult males collected in spring and late autumn (November), whereas butterflies of this form collected in early autumn (September and October) had few eupyrene sperm bundles (Table 6). On the other hand, summer-form butterflies had between 100 and 300 eupyrene sperm bundles at any one time over the season (Table 6).

**Figure 11:**
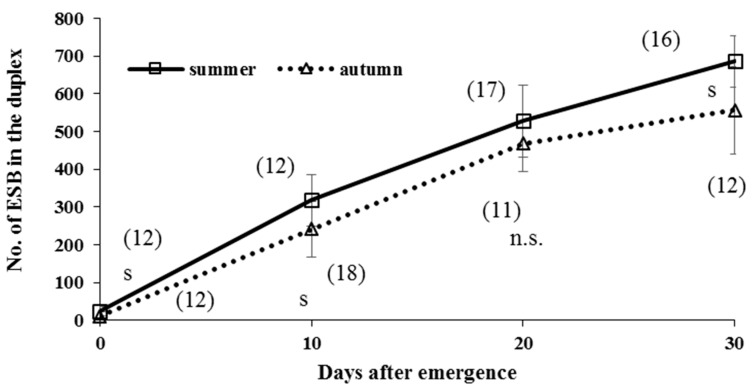
Changes of the number of eupyrene sperm bundles (ESB) in the duplex with adult age in summer- and autumn-form butterflies of *Polygonia c-aureum* under laboratory conditions. The number in the parenthesis indicates the sample size. S and n.s. indicate significant and not significant at *p* < 0.05, analyzed by Mann–Whitney *U* test, respectively.

In laboratory reared virgin butterflies, the number of eupyrene sperm bundles (ESB) in summer-form butterflies was significantly greater in autumn-form butterflies, at any adult age, except for day 20 (n = 28, *U1*-Value = 132.0, *U2*-Value = 55.0, *W*-Value = 121.0, *p* = 0.07) (Figure 11), probably due to the difference of body size. The number of ESB in the duplex of both seasonal forms increased with adult age.

### 3.8. Population Dynamics

**Figure 12:**
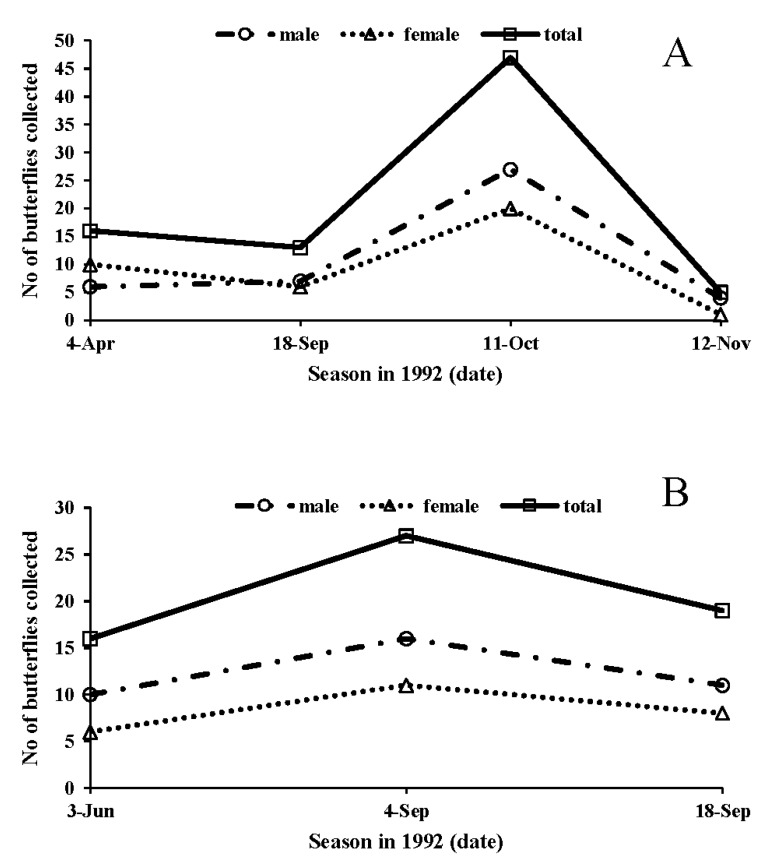
Population dynamics of autumn-form (**A**) or summer-form (B) of male, female or total (both sexes) *Polygonia c-aureum* butterflies over the various season in 1992.

Butterfly population size in the field study site was the greatest on 11 October in 1992 (Figure 12A). On that day, we tried to collect as many butterflies as possible, but we could not because the number was too great to examine in the laboratory. Thus, the real population size was much underestimated on that day. In fact, we found a lot of butterflies visiting nearby flowers to feed on nectar without mating. On 12 November, no butterflies were in flight, and the adult population size was very small, probably because autumn-form butterflies that had emerged in September or October were diapausing (hidden beneath the host plants) or had died. On the other hand, the number of summer-form butterflies was relatively high on the several summer sample dates (Figure 12B). Summer-form butterflies were often found near their host plant, flew or exhibited courting/mating behaviors. We did not observe autumn-form males exhibiting copulative behavior with summer-form females, or vice versa.

### 3.9. Phenology of the Host Plant

The main host plant of *P. c-aureum* in Japan is *Humulus japonicas* Siebold & Zucc (Japanese hop) (Cannabaceae). This plant is distributed across Japan, Taiwan, the Korean peninsula, China and the Indochina peninsula. Budding was observed from February to April at least in Saitama and Tokyo prefectures in Japan. Early budding sometimes caused the whole plant death because of frost or snow. This plant is a vine that spread over the ground, and covers other plants, resulting in the establishment of successful large colony. This plant started to bloom in September to October, differing in the region and site probably due to the differences of microclimate, quality of soil and/or strain. Blooming apparently worsened the quality of leaves eaten by *P. c-aureum* larvae, because larvae did not eat leaves with bad quality. Therefore, the bad quality of host plant would prolong the developmental period of larvae of this butterfly, especially in autumn, which would be relevant to determination of seasonal form. Blooming was induced by a short day-length from the observation conducted in the green house. Thus, illumination of telegraph pole suppressed the blooming. In late autumn, this plant was withered by the frost or snow, probably due to low temperature, in November to December. Interestingly, we found the grown colony of this plant in Chipon, Taiwan in March, indicating no death of them during the winter.

## 4. Discussion

In this study, we compared the laboratory results with those of field surveys on the development and reproduction in a nymphalid butterfly *Polygonia c-aureum*.

### 4.1. Spermatogenesis

Spermatogenesis did not fundamentally differ between non-diapausing summer-form and diapausing autumn-form males of *P. c-aureum* (Figure 7 and Figure 8) [112], except that autumn-form butterflies produced a small amount of both eupyrene and apyrene sperm after overwintering [113] and that summer-form butterflies cannot overwinter. Summer-form butterflies can use two types of sperm for insemination shortly after emergence. Although autumn-form butterflies also have an adequate quantity of sperm in the duplex shortly after emergence, they use sperm stored in the duplex before overwintering for mating in the following spring. Both seasonal forms of the butterfly are able to store eupyrene and apyrene sperm from the end of the pupal stage [88,114], and this sperm movement from the testis to the duplex continues to the adult stage (Figure 11). We found that neither photoperiod nor diapause affected spermatogenesis or sperm movement in this butterfly [78,113], and this finding could probably be applied to other adult-diapausing species, since adult spermatogenesis, especially eupyrene spermiogenesis, is inactive in lepidopteran species [112]. Most likely, autumn-form butterflies are able to protect sperm stored in the duplex during overwintering or adult diapause. Whether, in the study species, a portion of sperm stored in the duplex die or not during overwintering is unclear, but this is possible given that this phenomenon is known in several dipteran species [115,116,117,118]. If so, revival of spermatogenesis after overwintering may be important to renew numbers of apyrene and eupyrene sperm in the testis or duplex.

### 4.2. Development of Accessory Glands and Simplex

Under laboratory conditions, the development of accessory glands and simplex in *P. c-aureum* was suppressed by a short photoperiod and low temperatures in the adult stage before overwintering (Figure 5 and Figure 6). This reflects the fact that autumn-form butterflies emerging in autumn enter adult diapause. The simplex and accessory glands produce spermatophoral substances together with the sperm and semen derived from the testis, vas deferens, and duplex [119,120]. Our earlier study showed that development of the accessory gland and simplex is promoted by application of juvenile hormone analogue methoprene [42], suggesting that adult diapause of this butterfly is terminated by juvenile hormone. However, transplantation of active corpora allata that could produce and secrete the juvenile hormone into diapausing autumn-form of *P. c-aureum* adults did not stimulate mating behavior [111], suggesting that adult diapause in this butterfly is controlled by brain or other neural substances as well as the corpora allata.

### 4.3. Mating

Autumn-form *P. c-aureum* males can mate with autumn-form females in spring, but we did not observe any such matings in nature at any season, probably due to their specific behavior. In fact, autumn-form female butterflies collected in autumn did not have a spermatophore, suggesting that they do no mate before overwintering. Our results suggest that both females and males of this butterfly can copulate multiple times. Males mated approximately 5 times in their lives (mean for first 30 days of their adult life under laboratory researches), although mating frequency differs greatly among individuals. Since development of accessory glands and the simplex is suppressed in adult, diapausing autumn-form male butterflies are unable to produce spermatophores. This means that even if they were capable of mating, such matings would result in failure, with no passage of spermatophore to the female’s reproductive tract [121]. Mating failure is seen in males that have mated with a high frequency or with young females [43], but mating failure (in the sense of lack of female insemination) would rarely occur in nature in *P. c-aureum*, because of intense competition from other males and because of female choice. Spermatophore size in this butterfly was observed to increase with lower frequency of mating, and inter-mating intervals (Figure 3 and Figure 4). Spermatophore size declined with consecutive matings [24,43,56,57,122] and increased with age [123], although spermatophore size did not increase with age at first mating in this butterfly. The latter results suggest strongly that males of this butterfly have a limitation to accommodate the spermatophoral substances in their reproductive organs. In comparison, in the true armyworm, *Pseudaletia unipuncta* (Haw.), male reproductive success per mating remained relatively constant from the first to the sixth mating [124]. Small ejaculates in males that had mated multiple times may induce subsequent matings in females to re-mate with different males. To avoid or reduce the sperm competition, male butterflies transfer as large a spermatophore as possible or as many as possible, although male reproductive efforts are fundamentally limited by the supply of ejaculate components [55].

### 4.4. Spermatophore

In the European corn borer, *O. nubilalis*, the volume of spermatophore decreased exponentially with subsequent matings [125]. Similarly, multiple matings by adult males of this moth led to smaller spermatophores with too few sperm [125]. The present study on *P. c-aureum* showed similar results (Figure 4), which suggest that the ability of males to produce ejaculates deceases with repeated mating. Thus, an extreme decline in accessory glands and simplex secretions in contrast to levels in unmated adult males of the same or similar age may indicate frequent and/or recent mating. Although it may differ among species, ejaculate size is independent of male body size and female fecundity in the meadow grasshopper, *Chorthippus parallelus* (Zetterstedt) [126]. On the other hand, in the Mexican fruit fly, *Anastrepha ludens* Loew, female refractory period depends on male size and the nutritional condition of both sexes, suggesting that female receptivity does not depend only on the condition of the male ejaculate [47]. In the buff-tailed bumblebee, *Bombus terrestris* (L.), young or heavy males mate more swiftly and copulate for less time than old males or lighter males [127]. The forewing length of summer-form of *P. c-aureum* reared under a long photoperiod tended to be larger than that of autumn-form reared under a short photoperiod, coinciding with the results on pupal weight [100]. As body size often affects the size of reproductive organs, for example testis size, quantitative differences between summer- and autumn-form butterflies on testis size or the scale of spermatogenesis would be caused from these differences of body size.

### 4.5. Oogenesis and Mating Status in Females

Ovarian development in summer-form butterflies collected in the field proceeded, whereas that in autumn-form butterflies collected in autumn was completely suppressed (Table 2). The latter implies that autumn-form female butterflies were diapausing. Although we did not examine ovarian development in spring for overwintered autumn-form butterflies, it seems highly likely that they developed ovary, because they laid eggs onto the leaves of host plant in the observation separated from the present study. Mating rates of autumn-form female butterflies before overwintering were zero, indicating that they did not copulate in this season (Table 2). Mating rates of autumn-form after overwintering were 100%, and only one spermatophore was observed in the bursa of most females, suggesting that they were monoandry in spring. On the other hand, summer-form female butterflies had developed ovary and nearly half of them had several spermatophores in their bursa. These results suggest that summer-form female butterflies exhibit the tendency of polyandry, rather than monandry. If this is true, this butterfly may have different mating strategy between autumn-and summer-form females, depending on the season and physiological conditions, in which autumn-form female butterflies seen in spring might be willingness to engage in flight for intake of nectar or oviposition rather than multiple matings. Further studies are much needed to test these hypotheses.

### 4.6. Changes of Color or Size of Male Reproductive Organs

In general, any feature that can easily separate virgin from mated males would be useful in many kinds of studies on insect reproduction. For example, the age of wild-caught males was estimated from the number of eupyrene sperm bundles in the duplex of the tobacco budworm *H. virescens* (F.), although in several males the number of sperm bundles was too variable (from a few to large number) to be of practical use to assess mating status [128,129]. In the Mexican rice borer, *Eoreuma loftini* (Dyar) the best indicator of male mating status is the color of the duplex, although the color might lead to underestimates of the number of matings [130]. In the tobacco cutworm, *S. litura* (Fabricius), the color of the simplex changes from the colorless or pale yellow within 24 hr to orange several days later following mating [131]. Similarly, in the fall armyworm, *Spodoptera frugiperda* (J. E. Smith), the color of the simplex changes from light brown to black near the twisted portion to transparent to yellow after mating [132]. These authors mention that the change in color of the simplex can be used to determine mating status of the male, although the method requires consideration of moth age and post-mating time to obtain absolute accuracy. They suggest that relative age can be determined by fat body appearance and general moth condition. In *S. littoralis*, the changes in the color of the secretions in the simplex is sufficient to determine the mating status of field-caught males, although it may be less effective in old males [133]. For this reason, the width of the simplex is also used as a quantitative character to differentiate mated males from virgins in *S. litura* [134]. Similarly, in males of the butterfly *D. plexippus* weights of the dissected-out simplex are used to determine diapause status [135].

### 4.7. Estimation of Male Mating Status

Here, we propose a novel method for determining mating status of males of some butterflies, which would allow us to estimate the age of wild-caught butterflies and from age infer their mating status. For the approach, the first step is to categorize the reproductive development of an individual male butterfly using the following index. First, testis size was divided into four levels using the relationships shown in Figure 4, which indicates the following groups: 15.1 or above mm^3^ (VY: very young), 10.1~15 mm^3^ (Y: young), 5.1~10.0 mm^3^ (Y or O: young~old), or ~5.0 mm^3^ (VO: very old). Next the weight of the accessory glands was divided into three groups using the results shown in Figure 5, giving these groupings: for the summer-form: ~0.49 mg (VY), 0.5~0.69 mg (Y), 0.7 or above mg (O), and for the autumn-form, ~0.2 mg (VY), 0.21~0.6 mg, (Y or O), 0.61 or above mg (VO). Third, the weight of the simplex was divided into three groups based on findings in Figure 6, giving these groupings: for the summer form, ~3.5 mg (VY:), 3.6~5 mg (Y), 5.1 or above mg (O), and for the autumn form, ~1.5 mg (VY): 1.6~3.5 mg, (Y or O), 3.6 or above mg (VO). Fourth, the number of eupyrene sperm bundles (ESB) in the duplex was divided into three groups using the data from Figure 11 giving one set of values for both seasonal forms of the butterfly: ~200 (VY), 201~500 (Y or O) and 501 or above (VO). To apply the method, first the physiological age of wild-caught male butterflies was estimated based on the testis size [88]. If the index of the accessory gland, simplex or the number of eupyrene sperm bundles contradicts with the index of testis size, the male is presumed to have mated (See Table 7, Table 8 and Table 9). For example, if old adult males, having a small testis, have light accessory glands and simplex, they would be mated, because unmated old males could have heavy reproductive organs. As an exception, the results on the individual No. 254 were unclear, because the accessory gland did not develop (Table 8). We could not apply these criteria successfully to diapausing autumn- form butterflies, however, because old autumn-form males with a small testis have light accessory glands and simplex, suppressed by diapause, indicating the need for another set of criteria. Although the accuracy level of this approach has not yet been estimated across various species, the method has potential as an important new approach of general value. Similarly, age and mating status of wild-caught individuals of the latrine fly, *Fannia scalaris* F., were estimated from the testis size and other morphological characters in [136].

We estimated that the mating rate of overwintered autumn-form male butterflies of *P. c-aureum* in spring of 1991 and 1992 was about 46% (6/13 individual analyzed) (Table 7). If accurate, these results suggest that some overwintered male butterflies must mate multiple times (because most female butterflies were observed to be mated) (Table 2) because a proportion of males could not mate. Although we could not estimate mating status of pre-overwintering autumn-form butterflies using the present method, we believe most would be unmated because development of their accessory glands and simplex was suppressed, and all autumn-form females before overwintering were in reproductive diapause (Table 2). However, we could not exclude the possibility that a portion of autumn-form butterflies that emerged in September (early autumn) might mate with summer-form or autumn-form female butterflies in autumn. In fact, a similar case is known to the pierid butterfly *Eurema hecabe* (L.) [137]. On the other hand, the mating rate of summer-form male butterflies was estimated to be 100% (28/28 individuals) (Table 9). This suggests that all or most summer-form male butterflies could mate with summer-form females. In other words, most male butterflies in a population have the opportunity to mate, which suggests that autumn-form and summer-form male butterflies may have different mating strategies in the field like females.

## 5. Conclusions

In this study, we performed the field and laboratory examinations on the reproductive development and mating status in both sexes of a nymphalid butterfly, *Polygonia c-aureum*. It was found that diapausing generation (autumn-form) suppressed their ovarian development completely in the autumn, whereas non-diapausing generations (summer-form) progressed it in the summer. Female butterflies of summer-form showed the tendency of polyandry from the investigation of spermatophore in the bursa copulatrix, whereas those of overwintered autumn-form showed monoandry, suggesting that female butterflies may have a different mating strategy between non-diapausing and diapausing generations. We attempted to estimate the physiological age and mating status of adult males from the testis size and reproductive development. Testis shrank with increasing adult age. Thus, young male butterflies had large testis, whereas old butterflies had small testis. Furthermore, young male butterflies had light accessory glands and simplex, and less number of eupyrene sperm bundles in the duplex, whereas old butterflies exhibited the reversed results. Thus, we presumed, for example, if old adult males had light accessory glands and simplex as well as the small number of eupyrene sperm bundles in the duplex, they were judged as mated. On the contrary, if they had heavy accessory glands and simplex as well as the large amount of eupyrene sperm bundles, they were judged as unmated. As we could not apply these criteria to diapausing autumn-form butterflies, we should search for another set of criteria and further field investigations needed. We do hope that the present study will lead to understanding of male mating status in the field and of mating strategies in other lepidopteran species.

## Figures and Tables

**Table 1:** Development of oogenesis in *Polygonia c-aureum* females that are reared under a short or long photoperiod at 21 °C after emergence.

Photoperiod/Adult Age	No. of Insects Used	No. of Eggs (Mean ± S.D.)	Oocyte Diameter (mm) (Mean ± S.D.)
Short day			
0	9	0.00 ± 0.00	0.159 ± 0.019
10	40	0.00 ± 0.00 *	0.199 ± 0.014
20	32	0.00 ± 0.00 *	0.187 ± 0.029 *
30	26	0.00 ± 0.00 *	0.148 ± 0.011 *
40	20	0.80 ± 0.60 *	0.294 ± 0.059 *
50	21	10.20 ± 5.20 *	0.331 ± 0.059 *
60	19	9.80 ± 5.00 *	0.400 ± 0.057 *
Long day			
10	29	3.70 ± 2.00	0.302 ± 0.041
20	24	22.30 ± 5.20	0.529 ± 0.045
30	9	46.00 ± 11.60	0.684 ± 0.041
40	8	27.50 ± 7.70	0.650 ± 0.043
50	14	46.40 ± 7.70	0.687 ± 0.023
60	12	58.70 ± 8.20	0.676 ± 0.029

Asterisks indicate the significant differences at each adult age between a short and long day-length by Mann–Whitney *U*-test.

**Table 2:** Field data of female reproduction in oogenesis and mating rate in *Polygonia c-aureum*.

Seasonal Form	Date of Collection	No. of Insects Used	No. of Insects with Egg in the Ovary (%)	Percentages of Females with Spermatophore
Summer	3-June-1992	6	-	83.3
Summer	4-September-1992	11	11, 100	100
Summer	18-September-1992	8	8, 100	100
Autumn	3-April-1992	5	-	100
Autumn	18-September-1992	6	0, 0	0
Autumn	11-October-1992	20	0, 0	0
Autumn	12-November-1992	1	0, 0	0

**Table 3:** Seasonal changes of spermatophore size at each mating number in *Polygonia c-aureum*.

Seasonal Form/Date	Site	No. of Animals Used	Spermatophore Size (mm^3^)			
			No. of Spermatophore (Mean ± S.D.) From the Newest To the Oldest			
			1	2	3	4
Autumn						
3-April-1992	Tokyo	6	53.9 ± 24.6 (6)	63.2 ± 50.9 (2)		
18-September-1992	Saitama	6				
11-October-1992	Saitama	20				
12-November-1992	Saitama	1				
Summer						
3-June-1992	Saitama	5	68.5 ± 26.6 (5)			
4-September-1992	Saitama	11	35.4 ± 22.4 ^a^ (11)	30.8 ± 13.0 ^b^ (7)	47.1 ± 21.3 ^a,c^ (7)	45.2 ± 22.1 ^a,b^ (3)
18-September-1992	Saitama	8	34.5 ± 22.9 (11)	25.1 ± 19.7 (6)	41.1 ± 25.5 (5)	

Different letters indicate the significant differences among the number of spermatophore of summer-form females collected on 4th September 1992. The number of parenthesis indicates the sample size. The number of 1 spermatophore indicates recent mating, while the number of 4 spermatophore the first mating.

**Table 4:** Mating frequency and dry weight or volume of spermatophore (SP) in *Polygonia c-aureum*.

	No. of Insects Used	Min	Max	Mean ± S.D.
Mating frequency	10	0	15	5.11 ± 4.81
Weight of SP (mg) per mating	10	0	3.55	1.93 ± 0.46
Volume of SP (mm^3^) per mating	10	0	123.76	59.69 ± 48.68
Total volume of SP (mm^3^)	10	0	288.04	209.45± 57.93

Ten males were paired with 10 virgin females for their first 30 days of adult life. SP indicates spermatophore.

**Table 5:** Changes of testis volume and weight of accessory gland and simplex of *Polygoniac-aureum* in various season in 1991 and 1992.

Date of Collection	Site of Collection	Testis Volume (mm^3^) (Mean ± S.D.)	Accessory Gland Dry Weight (mg) (Mean ± S.D.)	Simplex Dry Weight (mg) (Mean ± S.D.)
Autumn form				
22-April-1991	Akigase	6.51 ± 1.613 (4) *a	0.80 ± 0.356(4)a	4.00 ± 2.592(4)a
31-March-1992	Akigase	2.95 ± 1.493(4)a	0.48 ± 0.382(3)ab	3.13 ± 2.730(3)ab
4-April-1992	Akigase	4.17 ± 1.260(6)a	0.39 ± 0.163(6)b	2.65 ± 1.170(6)ab
4-September-1992	Akigase	13.19 (1)	1.60 (1)	
18-September-1992	Akigase	12.89 ± 4.079(7)b	0.20 ± 0.071(6)bc	1.83 ± 0.523b
11-October-1992	Akigase	13.13 ± 2.505(26)b	0.13 ± 0.094(27)c	1.90 ± 0.523(27)b
12-November-1992	Akigase	5.49 ± 0.950(4)a	0.20 ± 0.115(4)bc	2.15 ± 1.158(4)ab
Summer form				
13-June-1991	Akigase	13.12 ± 6.349(13)ab	0.58 ± 0.269(14)ab	3.76 ± 1.615(14)a
15-July-1991	Akigase	12.85 ± 3.115(12)ab	0.44 ± 0.152(5)ab	2.66 ± 0.826(5)ab
19-August-1991	Akigase	15.93 ± 4.259(12)ab	0.39 ± 0.272(12)ab	3.25 ± 1.566(12)ab
3-September-1991	Akigase	14.45 ± 5.575(4)b		1.83 ± 0.814(4)ab
3-June-1992	Akigase	12.08 ± 2.764(10)ab	0.70 ± 0.372(10)a	3.03 ± 1.231(10)ab
20-August-1992	Tsurugashima	12.62 ± 1.669(5)ab	0.48 ± 0.084(5)b	2.33 ± 1.060(5)ab
4-September-1992	Akigase	10.37 ± 2.659(16)abc	0.35 ± 0.246(16)bc	2.79 ± 1.407(16)ab
18-September-1992	Akigase	6.13 ± 1.489(11)c	0.30 ± 0.297(11)bc	1.76 ± 0.988(11)b

Different letters indicate the significant differences. The data was analyzed among each seasonal form by Tukey’s method after ANOVA. The number of parenthesis indicates the sample size.

**Table 6:** Changes of the number of eupyrene sperm bundles (ESB) of autumn- and summer- forms in the wild male *Polygonia c-aureum* butterflies over the season in 1991 and 1992.

Date of Collection	Site of Collection	No. of Insects Used	No. of ESB in the Duplex (Mean ± S.D.)
Autumn form			
22-April-1991	Akigase	4	312 ± 179.1abc
31-March-1992	Kominawa	3	427 ± 284.0b
4-April-1992	Akigase	6	481 ± 163.0b
18-September-1992	Akigase	4	105 ± 62.3a
11-October-1992	Akigase	7	86 ± 51ab
12-November-1992	Akigase	10	403 ± 166.4c
Summer form			
13-June-1991	Akigase	8	240 ± 206.2a
15-July-1991	Akigase	5	135 ± 52.2a
19-August-1991	Akigase	9	166 ± 124.8a
3-September-1991	Akigase	4	93 ± 79.6a
3-June-1992	Akigase	5	70 ± 28.4a
20-August-1992	Tsurugashima	5	266 ± 375.6a
4-September-1992	Akigase	9	214 ± 134.2a
18-September-1992	Akigase	9	274 ± 149.6a

Different letters indicate the significant differences. The data was analyzed among each seasonal form by Tukey’s method after ANOVA.

**Table 7:** The data on the volume or weight of each reproductive organ of wild caught autumn-form adult males of *Polygonia c-aureum*.

Date of Collection	Individual No.	Testis Size (mm^3^)	Accessory Gland Weight (mg)	Simplex Weight (mg)	No. of ESB in the Duplex	Justice Mated or Not
1991						
22-April	29	6.40 (O)	1.0 (VO)	6.4 (O)	550 (O)	unmated
	30	4.45 (VO)	0.5 (O)	2.4 (Y or O)	309 (Y or O)	mated
	31	6.84 (O)	0.5 (O)	1.2 (VY)	117 (VY)	mated
	32	8.37 (O)	1.2(VO)	6.0 (O)	273 (Y or O)	unmated
1992						
31-March	3415	1.86 (VO)	0.9 (VO)	5.6 (O)	754 (O)	unmated
	3416	2.34 (VO)	0.15 (VY)	0.2 (VY)	245 (Y or O)	mated
	3417	0.84 (VO)	0.4 (O)	3.6 (Y or O)	281 (Y or O)	mated
4-April	17	1.18 (VO)	0.4 (O)	1.2 (VY)	325 (Y or O)	mated
	18	2.34 (VO)	0.7 (VO)	4.7 (O)	769 (O)	unmated
	19	2.38 (VO)	0.25 (Y or O)	2.6 (Y or O)	476 (Y or O)	mated
	20	2.90 (VO)	0.4 (Y or O)	3.1 (Y or O)	500 (Y or O)	unmated
	21	1.51 (VO)	0.3 (Y or O)	2.5 (Y or O)	498 (Y or O)	mated
	22	2.22 (VO)	0.3 (Y or O)	1.9 (Y or O)	323 (Y or O)	mated

VY, Y, O, and VO indicate very young, young, old, and very old, respectively. If the index of accessory. gland, simplex, or the number of eupyrene sperm bundles (ESB) contradicts with the index of testis, it was judged to be mated.

**Table 8:** The data on the volume or weight of each reproductive organ of wild caught summer-form adult males of *Polygonia c-aureum* in 1991.

Date of Collection	Individual No.	Testis Size (mm^3^)	Accessory Gland Weight (mg)	Simplex Weight (mg)	No. of ESB in the Duplex	Justice Mated or Not
13-June	247	4.34 (VO)	0.6 (Y)	3.9 (Y or O)	294 (Y or O)	mated
	248	3.29 (VO)	0.3 (VY)	2.4 (VY)	224 (Y or O)	mated
	249	3.55 (VO)	0.8 (O)	4.9 (Y or O)	496 (Y or O)	unmated
	250	5.72 (O)	0.7 (O)	4.8 (Y or O)	87 (VY)	mated
	251	4.03 (VO)	0.2 (VY)	1.3 (VY)	43 (VY)	mated
	252	11.43 (Y)	0.4 (VY)	2.4 (VY)	81 (VY)	unmated
	253	12.58 (Y)	0.8 (O)	5.2 (Y or O)	102(VY)	mated
	254	4.50 (VO)	0.2 (VY)	6.9 (O)	590 (O)	unmated?
15-July	695	7.00 (O)	0.6 (Y or O)	3.8 (Y or O)	140 (VY)	mated
	696	9.31 (O)	0.4 (VY)	1.7 (VY)	104 (VY)	mated
	697	10.02 (Y)	0.6 (Y)	3.1 (VY)	148 (VY)	unmated
	698	6.07 (O)	0.3 (VY)	2.6 (VY)	72 (VY)	mated
	699	5.41 (O)	0.3 (VY)	2.1(VY)	211 (Y or O)	mated
19-August	1068	9.82 (O)	1.1 (O)	6.3 (O)	191 (VY)	mated
	1069	9.54 (O)	0.3 (VY)	4.5 (Y or O)	79 (VY)	mated
	1070	10.37 (Y)	0.4 (VY)	4.1 (Y or O)	105 (VY)	unmated
	1071	7.22 (O)	0.25 (VY)	1.4 (VY)	218 (Y or O)	mated
	1072	8.30 (O)	0.5 (Y)	2.6 (VY)	78 (VY)	mated
	1073	4.34 (VO)	0.5 (Y)	3.2 (VY)	457 (Y or O)	mated
	1074	11.50 (Y)	0.4 (VY)	5.1 (Y or O)	151 (VY)	unmated
	1075	4.87 (VO)	0.1 (VY)	1.8 (VY)	179 (VY)	mated
	1076	7.58 (O)	0.1 (VY)	1.1 (VY)	35 (VY)	mated
3-September	1359	10.35 (Y)	0.5 (Y)	2.9 (VY)	94 (VY)	unmated
	1360	4.16 (VO)	0.1 (VY)	1.1 (VY)	204 (Y or O)	mated
	1361	8.63 (O)	0.1 (VY)	2.0 (VY)	26(VY)	mated
	1362	5.76 (O)	0 (VY)	1.3 (VY)	46 (VY)	mated

VY, Y, O, and VO indicate very young, young, old, and very old, respectively. If the index of accessory gland, simplex, or the number of eupyrene sperm bundles (ESB) contradicts with the index of testis, it was judged to be mated.

**Table 9:** The data on the volume or weight of each reproductive organ of wild caught summer-form adult males of *Polygonia c-aureum* in 1992.

Date of Collection	Individual No.	Testis Size (mm^3^)	Accessory Gland Weight (mg)	Simplex Weight (mg)	No. of ESB in the Duplex	Justice Mated or Not
3-June	246	7.54 (VO)	0.48 (VY)	4.8 (Y or O)	99 (VY)	mated
	247	5.04 (VO)	0.55 (Y)	4.45 (Y or O)	70 (VY)	mated
	248	4.19 (VO)	0.28 (VY)	1.7 (VY)	42 (VY)	mated
	249	7.94 (VO)	0.64 (Y)	4.4 (Y or O)	103 (VY)	mated
	250	7.25 (VO)	0.28 (VY)	1.7 (VY)	35 (VY)	mated
20-August	944	5.95 (VO)	0.4 (VY)	1.05 (VY)	147 (VY)	mated
	946	6.81 (VO)	0.6 (Y)	3.5 (VY)	78 (VY)	mated
	947	5.62 (VO)	0.5 (Y)	3.3 (VY)	78 (VY)	mated
	948	7.46 VO)	0.5 (Y)	2.2 (VY)	93 (VY)	mated
	949	5.74 (VO)	0.4 (VY)	1.6 (VY)	91 (VY)	mated
4-September	1134	8.56 (O)	0.1 (VY)	1.5 (VY)	95 (VY)	mated
	1135	5.52 (VO)	0.5 (Y)	5.0 (Y or O)	272 (Y)	mated
	1136	5.10 (VO)	0.1 (VY)	2.2 (VY)	93 (VY)	mated
	1137	5.22 (VO)	0.5 (Y)	2.8 (VY)	506(O)	mated
	1138	5.16 (VO)	0.5 (Y)	2.95 (VY)	269 (Y)	mated
	1139	4.21 (VO)	0.9 (O)	4.7 (Y or O)	165 (VY)	mated
	1144	4.18 (VO)	0.1 (VY)	1.4 (VY)	79 (VY)	mated
	1146	5.13 (VO)	0.3 (VY)	2.45 (Y or O)	263 (Y)	mated
	1147	3.44 (VO)	0.4 (VY)	2.5 (Y or O)	188 (VY)	mated
18-September	1402	3.43 (VO)	0.2 (VY)	1.9 (VY)	277 (Y)	mated
	1412	2.47 (VO)	0.4 (VY)	1.85 (VY)	446 (Y)	mated
	1424	2.03 (VO)	0.1 (VY)	1.9 (VY)	500 (Y)	mated
	1426	4.21 (VO)	0 (VY)	1.6 (VY)	195(VY)	mated
	1427	3.97 (VO)	0.8 (O)	4.4 (Y or O)	383 (Y)	mated
	1428	3.52 (VO)	0 (VY)	0.6 (VY)	9 (VY)	mated
	1429	3.40 (VO)	0.2 (VY)	1.1 (VY)	236 (Y)	mated
	1430	2.18 (VO)	0.3 (VY)	1.3 (VY)	195 (VY)	mated
	1431	3.47 (VO)	0.15 (VY)	1.2 (VY)	222 (Y)	mated

VY, Y, O, and VO indicate very young, young, old, and very old, respectively. If the index of accessory gland, simplex, or the number of eupyrene sperm bundles (ESB) contradicts with the index of testis, it was judged to be mated.
